# Mitochondrial Reactive Oxygen Species Contribute to Pathological Inflammation During Influenza A Virus Infection in Mice

**DOI:** 10.1089/ars.2019.7727

**Published:** 2020-03-24

**Authors:** Eunice E. To, Jonathan R. Erlich, Felicia Liong, Raymond Luong, Stella Liong, Farisha Esaq, Osezua Oseghale, Desiree Anthony, Jonathan McQualter, Steven Bozinovski, Ross Vlahos, John J. O'Leary, Doug A. Brooks, Stavros Selemidis

**Affiliations:** ^1^ Program in Chronic Infectious and Inflammatory Diseases, Oxidant and Inflammation Biology Group, School of Health Sciences and Biomedical Sciences, College of Science, Engineering & Health, RMIT University, Melbourne, Australia.; ^2^ Infection and Immunity Program, Department of Pharmacology, Biomedicine Discovery Institute, Monash University, Melbourne, Australia.; ^3^ Department of Histopathology Trinity College Dublin, Sir Patrick Dun's Laboratory, Central Pathology Laboratory, St James's Hospital, Dublin, Ireland.; ^4^ Molecular Pathology Laboratory, Coombe Women and Infants' University Hospital, Dublin, Ireland.; ^5^ Division of Health Sciences, School of Pharmacy and Medical Sciences, Sansom Institute for Health Research, University of South Australia, Adelaide, Australia.

**Keywords:** mitochondria, reactive oxygen species, influenza, lung inflammation

## Abstract

***Aims:*** Reactive oxygen species (ROS) are highly reactive molecules generated in different subcellular sites or compartments, including endosomes *via* the NOX2-containing nicotinamide adenine dinucleotide phosphate oxidase during an immune response and in mitochondria during cellular respiration. However, while endosomal NOX2 oxidase promotes innate inflammation to influenza A virus (IAV) infection, the role of mitochondrial ROS (mtROS) has not been comprehensively investigated in the context of viral infections *in vivo*.

***Results:*** In this study, we show that pharmacological inhibition of mtROS, with intranasal delivery of MitoTEMPO, resulted in a reduction in airway/lung inflammation, neutrophil infiltration, viral titers, as well as overall morbidity and mortality in mice infected with IAV (Hkx31, H3N2). MitoTEMPO treatment also attenuated apoptotic and necrotic neutrophils and macrophages in airway and lung tissue. At an early phase of influenza infection, that is, day 3 there were significantly lower amounts of IL-1β protein in the airways, but substantially higher amounts of type I IFN-β following MitoTEMPO treatment. Importantly, blocking mtROS did not appear to alter the initiation of an adaptive immune response by lung dendritic cells, nor did it affect lung B and T cell populations that participate in humoral and cellular immunity.

***Innovation/Conclusion:*** Influenza virus infection promotes mtROS production, which drives innate immune inflammation and this exacerbates viral pathogenesis. This pathogenic cascade highlights the therapeutic potential of local mtROS antioxidant delivery to alleviate influenza virus pathology.

## Introduction

Influenza A virus (IAV) infections are responsible for annual epidemics and sporadic pandemics, which cause very significant respiratory illness that is a major burden for patients and global health care systems (11, 38). The present strategies to combat IAV are preventative vaccines and therapeutic antiviral drugs. However, the efficacy of vaccines for preventing the infection and containing the spread of IAV is suboptimal, mainly due to antigenic variance, which leads to mismatches between the vaccine and circulating strains and this reduces the effectiveness of vaccination approaches (16, 23). Moreover, there is a narrow window of therapeutic efficacy for antiviral drugs, and the emergence of drug-resistant viral strains also tends to render these antivirals ineffective (17, 26). Thus, there is an unmet need for effective viral therapeutics and one attractive option is to develop novel therapeutics that target critical viral biology that is independent of the strain and pathogenicity.

**Innovation**

Emerging evidence has implicated mitochondrial reactive oxygen species (mtROS) in regulating the immune responses to viral and bacterial infections. However, the role of mtROS in the context of influenza virus infections *in vivo* has not yet been investigated. This is the first study to highlight the therapeutic potential of mitochondria-targeted inhibition of the oxidative stress *in vivo* to alleviate the clinical symptoms and lung inflammation during influenza A virus infection. This application may afford protection against other debilitating diseases in humans that cause high oxidative environments, including chronic inflammatory disorders such as gout and cancer.

Reactive oxygen species (ROS) are a family of oxygen-containing molecules that are generated either deliberately by dedicated enzymes such as the nicotinamide adenine dinucleotide phosphate (NADPH) oxidase enzyme family or as a consequence of cellular metabolism from the mitochondria. ROS play diverse roles in physiology and pathophysiology, including in cell signaling, cell proliferation and apoptosis. The prototypical role of ROS in the regulation of the immune system and, for example, the killing of phagocytosed bacteria by NOX2-containing NADPH oxidase-dependent ROS production are clearly evident. During viral infection, there is also rapid generation of ROS, but paradoxically IAV infection promotes oxidative stress-dependent cellular damage and lung injury (5, 18, 30, 49). However, the specific subcellular localization and sources of ROS production that exert these detrimental effects are only starting to become evident. Cells have evolved ways to compartmentalize ROS production to minimize inadvertent side effects of ROS action on critical cellular machinery. The subcellular site of ROS generation does, however, tend to govern their biological action due to their highly diffusion limited nature (34). We have recently shown that viruses, including seasonal and pandemic influenza that enter cells by endocytosis, activate the NOX2-containing NADPH oxidase that generates ROS within endosomes (44). Furthermore, we revealed that the spatially restricted generation of H_2_O_2_ within endosomes during IAV internalization actually suppressed TLR7 activity located on the endosomal membrane. We also developed an innovative molecular targeting system to deliver a specific NOX2 oxidase inhibitor to dampen this response and limit viral pathogenesis (33). This innovative site-specific targeting system provided proof-of-principle for limiting ROS production to restrict viral pathogenesis (44).

It is well established that invading pathogens often drive alterations in cellular metabolism and specifically, mitochondrial function. For example, in macrophages, lipopolysaccharide drives a critical switch from oxidative phosphorylation to glycolysis, resulting in significant mitochondrial ROS (mtROS) generation (20, 27). Recently, mtROS have been implicated in the regulation of innate immune responses following bacterial and viral infections (21, 53), and while this response is likely to be complex, there is clear evidence of mtROS impacting on inflammasome activation. For example, mtROS can directly activate the NACHT, LRR, and PYD domain-containing protein 3 (NLRP3) inflammasome, leading to caspase 1 activation and subsequent maturation and release of the proinflammatory mediator IL-1β (28, 46); this is involved in the inflammatory immune response to IAV infection (19). Consequently, mice that are genetically deficient in components of the NLRP3 inflammasome complex have an effective immune response that is protective against highly pathogenic IAV strains (2, 42). The NLRP3 inflammasome was also shown to be necessary for the production of IL-1β in the lungs that was driving airway inflammation and protecting against lung pathology. In addition, the PB1-F2 peptide of pandemic viral strains such as PR8 and H7N9 induced a robust mtROS response (25). Inhibition of this mtROS with MitoTEMPO reduced the proteolytic maturation of both IL-1β and caspase 1, and IL-1β secretion in isolated mouse and human macrophages (32). Although early inhibition of NLRP3 inflammation with MCC950 exacerbates IAV disease severity in mice, late inhibition also alleviated disease and reduced lung inflammation (41). Overall, it appears that the inflammasome plays a dual role in the pathogenesis of IAV infection, but it is clear that mtROS can have a direct impact on immune function and viral pathogenesis.

To the best of our knowledge, the effect of inhibiting mtROS production on IAV pathogenesis has not been investigated *in vivo*. In addition, given that NOX2-derived endosomal ROS negatively regulate type I IFN production to IAV (44), we hypothesized that scavenging mtROS could also influence type I IFN production. Therefore, in the present study, we examined the role of mtROS in modulating the early innate and subsequent adaptive immune responses in an *in vivo* mouse model of IAV infection, and in particular assessed the effect on type I IFN and IL-1β expression, as well several other markers of lung inflammation, oxidative stress, and disease severity. To investigate this potential role of mtROS in influenza pathogenesis, we made use of intranasal delivery of the mtROS-specific scavenger MitoTEMPO in our well-established infection model. We have shown that MitoTEMPO treatment 1 day before IAV infection significantly reduced macrophage mtROS and lung IL-1β production, and this was associated with reduced mortality, airway and lung inflammation, as well as oxidative stress at both early and later stages of infection. Critically, MitoTEMPO significantly increased early type I IFN production and this was associated with a marked reduction in lung viral titers and enhanced resolution of proinflammatory cytokines at late stages of infection. These data support the idea that excessive mtROS can promote a deleterious early innate inflammatory immune response to influenza virus infection, which may be exploited as a novel therapeutic target *via* local delivery of mtROS scavengers.

## Results

### MitoTEMPO reduces IAV-induced body weight loss, mortality, and airway inflammation

**FIG. 1.**
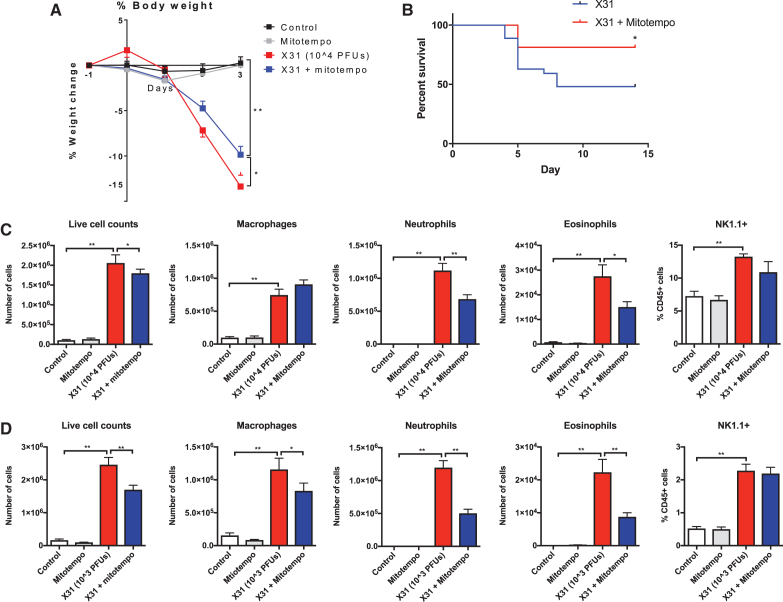
**Intranasal delivery of MitoTEMPO reduces IAV-induced body weight loss, mortality, and airway inflammation.** C57Bl/6J mice were treated once daily *via* intranasal administration of MitoTEMPO (100 μg) over a 4–6-day period 1 day before virus infections. Mice were intranasally infected with X31 **(**10^4^ PFUs in **C** or 10^3^ PFUs in **D)** or PBS control. **(A, B)** Daily body weight measurements were recorded over the 4–6-day period. Airway inflammation was assessed *via* counting the total number of live cells that were differentiated into macrophages, neutrophils, and eosinophils at **(C)** day 3 p.i and **(D)** day 5 p.i. Five hundred cells were counted from random fields by standard morphological criteria. In addition, NK cells (NK1.1^+^) were measured by flow cytometry in cells isolated from the lung. Data are expressed as mean ± SEM (Control, *n* = 10–12; MitoTEMPO, *n* = 10–12; X31 *n* = 18–20 X31+mito *n* = 18–20). Statistical analysis was conducted using a two-way ANOVA followed by Holm Sidak's *post hoc* multiple comparison test in **(A)** and one-way ANOVA test followed by Tukey's *post hoc* test for multiple comparison test for **(C, D)**. Statistical significance was taken where *p* < 0.05 (*p < 0.05 and **p < 0.01). For **(B)**, a Kaplan–Meier curve analysis was performed. Statistical significance was taken where *p* < 0.05. ANOVA, analysis of variance; IAV, influenza A virus; PBS, phosphate-buffered saline; PFU, plaque forming unit; SEM, standard error of the mean.

Body weight measurements were recorded daily as a surrogate index of disease severity. Animals that were infected with 10^4^ plaque forming units (PFUs) of IAV had lost ∼14% of their body weight at 3 days postinfection. IAV mice that were treated with MitoTEMPO lost significantly less of their total body weight in comparison with the virus-infected vehicle-treated group (Fig. 1A). Moreover, mice treated with MitoTEMPO showed a significant increase in survival rate (Fig. 1B). Importantly, MitoTEMPO-treated naive mice did not lose any body weight over the course of the experiment (Fig. 1A).

To evaluate airway inflammation, the total number of live cells in the bronchoalveolar lavage (BAL) was counted. Intranasal challenge with Hkx-31 IAV caused an increase in airway inflammation at day 3 and 5 postinfection (Fig. 1C, D, respectively). This response was reduced with MitoTEMPO treatment at day 3 and was further decreased at day 5 postinfection. Infection with IAV caused a rapid infiltration of circulating neutrophils, macrophages, lymphocytes, eosinophils, and natural killer cells (Fig. 1C, D). MitoTEMPO treatment caused a marked 40%–60% reduction in neutrophil cell counts and a 50%–60% reduction in eosinophil cell counts at both of these time points (Fig. 1C, D). At day 3, the number of macrophages and natural killer cells was also unaltered by MitoTEMPO treatment, but there was a significant reduction in BAL fluid (BALF) macrophages at day 5 (Fig. 1D). Importantly, the effect of MitoTEMPO administration appeared to be localized to the lung and did not modify the systemic inflammatory response (Supplementary Fig. S1).

### MitoTEMPO abrogated mtROS

**FIG. 2.**
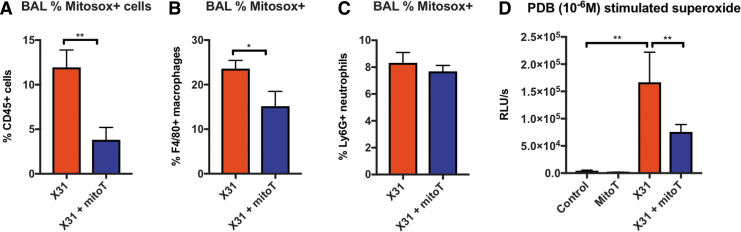
**Intranasal treatment with MitoTEMPO reduces mitochondrial and NOX2-derived ROS generation at day 3 postinfection.** C57Bl/6J mice were treated once daily *via* intranasal administration of MitoTEMPO (100 μg) over a 4-day period 1 day before virus infections. Mice were intranasally infected with X31 (10^4^ PFUs) or PBS control. At day 3 p.i, BALF was collected for **(A)** mitochondrial ROS measured by staining cells with a fluorescent probe (MitoSOX) and assessed using flow cytometric analysis. The populations are measured as a percentage of MitoSOX^+^ cells gated from the CD45^+^ population. **(B)** MitoSOX^+^ cells were further characterized in CD45^+^ F4/80^+^ macrophages and **(C)** CD45^+^ Ly6G^+^ neutrophils. **(D)** PDB (10^−6^ *M*) stimulated ROS production that was quantified by L-O12-enhanced chemiluminescence from BALF inflammatory cells. Data are expressed as mean ± SEM (Control, *n* = 6–8; MitoTEMPO, *n* = 6–8; X31 *n* = 10 X31+mito *n* = 12–14). Statistical analysis was conducted using **(A–C)** Students' unpaired *t*-test and in **(D)** one-way ANOVA test followed by Tukey's *post hoc* test for multiple comparisons. Statistical significance was taken where *p* < 0.05 (*p < 0.05 and **p < 0.01). BALF, bronchoalveolar lavage fluid; PDB, phorbol dibutyrate; ROS, reactive oxygen species.

To determine the effectiveness of MitoTEMPO at suppressing mtROS, we measured MitoSOX^+^ cells in the BAL and lung using flow cytometric analysis. At day 3 postinfection, ∼12% of the CD45^+^ cells in the BAL of virus-infected mice stained positively for MitoSOX (Fig. 2A). This response was significantly reduced with MitoTEMPO treatment, indicating that MitoTEMPO works effectively to inhibit mtROS at the direct site of inhibitor administration (*i.e.*, in the lungs). Furthermore, the mtROS response was blunted in the macrophage (Fig. 2B), but not the neutrophil population (Fig. 2C).

L-012-enhanced chemiluminescence was used to measure NOX2 oxidase-dependent ROS generation in BAL isolated from mice. BAL inflammatory cells taken from naive mice treated with MitoTEMPO had similar levels of ROS to the control group (Fig. 2D). BAL cells retrieved from IAV-infected mice exhibited a significant increase in ROS production compared with the uninfected control cohort, but this was significantly blunted with mitoTEMPO treatment at day 3 p.i (Fig. 2D). In isolated RAW 264.7 macrophages, MitoTEMPO treatment had no effect on the NOX2 oxidative burst (Supplementary Fig. S2), and thus, MitoTEMPO does not possess nonspecific inhibitory effects against NOX2 activity.

### Reduction in viral mRNA with MitoTEMPO treatment

**FIG. 3.**
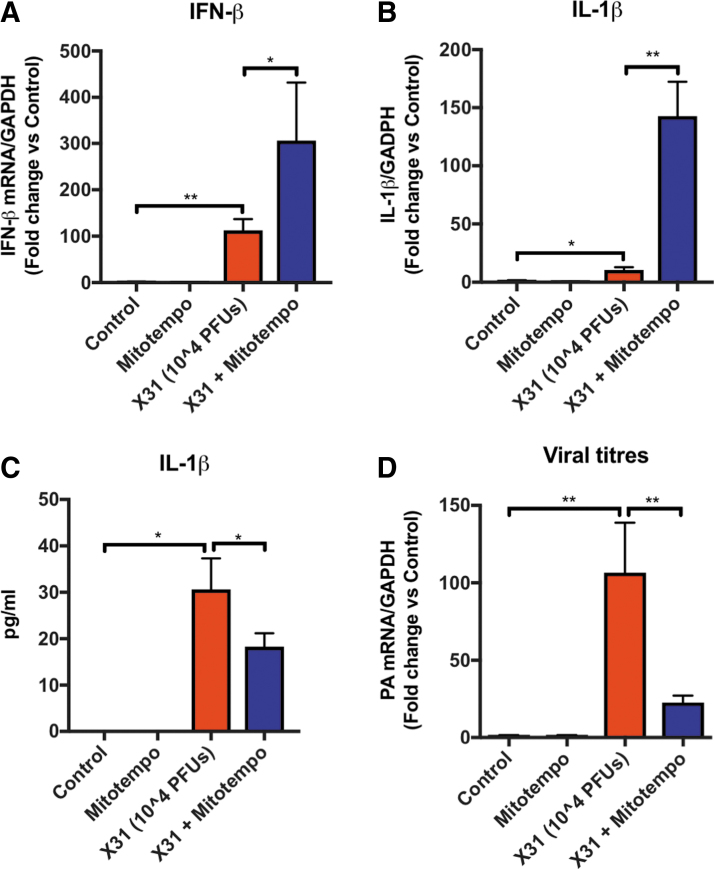
**Cytokine and chemokine expression at early phases of IAV infection with MitoTEMPO treatment.** Mice were infected with Hkx-31 at (10^4^ PFUs) or PBS (control) *via* intranasal administration. Animals were treated once daily *via* intranasal administration of MitoTEMPO (100 μg) over a 4-day period 1 day before virus infections for measurements at day 3 of **(A)** IFN-β and **(B)** IL-1β mRNA expression with qPCR; **(C)** IL-1β protein by ELISA and **(D)** mRNA from the gene encoding polymerase of influenza virus strain by qPCR in lung tissue. Responses are relative to GAPDH and then expressed as a fold-change above naive controls. Data are expressed as mean ± SEM (Control, *n* = 6–8; MitoTEMPO, *n* = 6–8; X31 *n* = 10 X31+mito *n* = 12–14). Statistical analysis was conducted using one-way ANOVA test followed by Tukey's *post hoc* test for multiple comparisons. Statistical significance was taken where *p* < 0.05 (*p < 0.05 and **p < 0.01). ELISA, enzyme-linked immunosorbent assay; GAPDH, glyceraldehyde 3-phosphate dehydrogenase; qPCR, quantitative polymerase chain reaction.

The expression of type I IFN-β and IL-1β was determined by quantitative polymerase chain reaction (qPCR) using lung tissue from uninfected control mice and mice infected with IAV. The expressions of both IFN-β and IL-1β were significantly elevated at day 3 post-IAV infection compared with the naive controls (Fig. 3A, B). In mice treated with MitoTEMPO, the expression of IFN-β and IL-1β transcript was significantly (∼3- and ∼10-fold, respectively) higher than the virus-infected control groups (Fig. 3A, B) at day 3. MitoTEMPO treatment in naive uninfected mice had no effect on baseline levels of IFN-β or IL-1β measured. In striking contrast, the amount of IL-1β protein in the BALF was significantly reduced by MitoTEMPO treatment (Fig. 3C). We next sought to examine the viral load in the lungs by analyzing the mRNA from the gene encoding segment 3 polymerase of IAV. At day 3 post-IAV infection, the presence of IAV mRNA was significantly lower in mice treated with MitoTEMPO, indicating that mtROS promote virus pathogenicity (Fig. 3D). Importantly, on day 5 post-IAV infection, IFN-β, IFN-γ, IL-1β, IL-6, TNF-α, IL-17A, CXCL2, and CCL3 expression in the lung was significantly lower in mice treated with MitoTEMPO and this was associated with significantly lower levels of influenza polymerase (PA) mRNA expression (Supplementary Fig. S3). Therefore, MitoTEMPO treatment accelerated the resolution of proinflammatory and antiviral cytokine expression and this was associated with reduced neutrophilia and airway inflammation.

### MitoTEMPO attenuated pulmonary inflammation

**FIG. 4.**
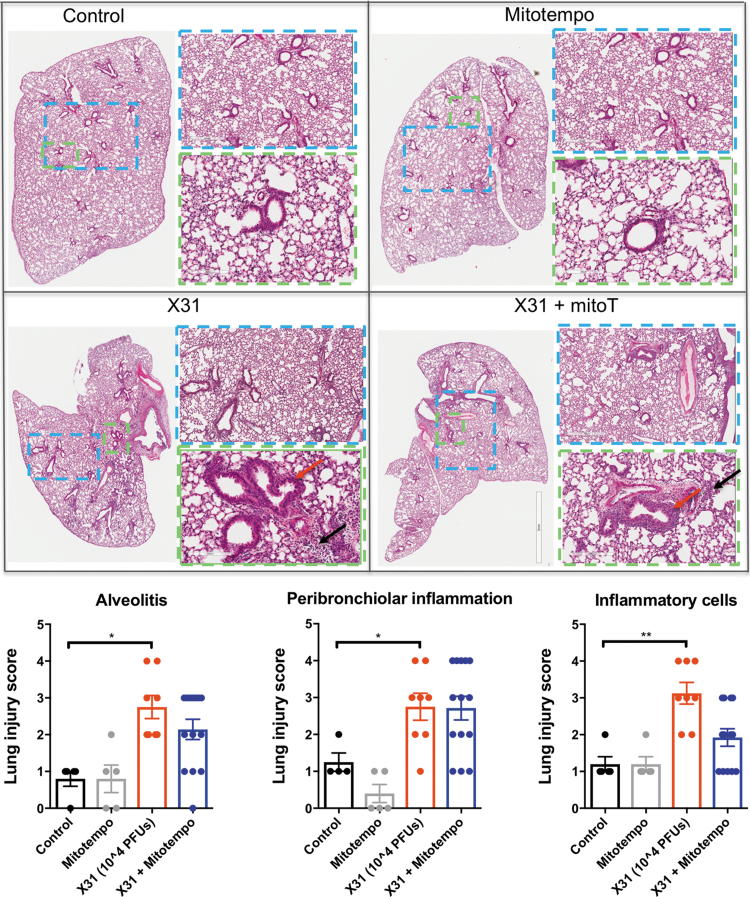
**MitoTEMPO relieves lung histopathological changes in Hkx-31-infected mice.** Histopathological analysis of lungs from C57Bl/6J mice treated once daily *via* intranasal administration of MitoTEMPO (100 μg) over a 4-day period 1 day before virus infections. Mice were intranasally infected with X31 (10^4^ PFUs) or PBS (control). Representative images displaying the inflammation in lungs that were sectioned longitudinally following H&E staining at day 3 postinfection. Each sample was scored blindly from 0 to 5 for each individual mouse (higher numbers indicate increased severity) from two independent assessors. Sections were scored for alveolitis (*black arrows*), inflammatory cell infiltrate, and peribronchiolar inflammation (*red arrows*). Representative images are presented at three different magnifications (1 × [clear], 3 × [*blue*], 6 × [*green*]). Data are expressed as mean ± SEM (Control, *n* = 6–8; MitoTEMPO, *n* = 6–8; X31 *n* = 10 X31+mito *n* = 12–14). Statistical analysis was conducted using one-way ANOVA test followed by Tukey's *post hoc* test for multiple comparisons. Statistical significance was taken where *p* < 0.05 (*p < 0.05 and **p < 0.01). H&E, hematoxylin and eosin.

**FIG. 5.**
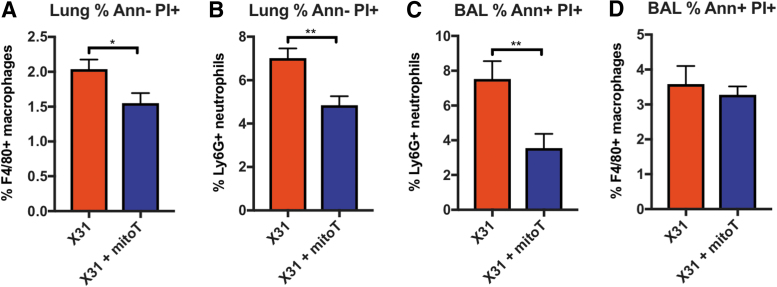
**MitoTEMPO reduces markers of apoptosis and necrosis in macrophages and neutrophils.** C57Bl/6J mice were treated once daily *via* intranasal administration of MitoTEMPO (100 μg) over a 4-day period 1 day before virus infections. Mice were intranasally infected with X31 (10^4^ PFUs) or PBS control. Apoptotic markers were measured at day 3 p.i by staining with Ann and PI in cells isolated from the lung and BAL and assessed using flow cytometric analysis. Characterization of apoptosis was carried out in macrophages and neutrophils as follows: **(A)** F4/80^+^Ann^−^ PI^+^ (necrosis), **(B)** Ly6G^+^Ann^−^ PI^+^ (necrosis), **(C)** Ly6G Ann^+^ PI^+^ (late apoptotic), and **(D)** F4/80^+^Ann^+^ PI^+^ (late apoptotic). Data are expressed as mean ± SEM (Control, *n* = 4; MitoTEMPO, *n* = 4; X31 *n* = 6 X31+mito *n* = 6). Statistical analysis was conducted using a Students' unpaired *t*-test. Statistical significance was taken where *p* < 0.05 (*p < 0.05 and **p < 0.01). Ann, Annexin V; BAL, bronchoalveolar lavage; PI, propidium iodide.

Cellular infiltration in the lung and alveolar space is a major contributing factor to IAV pathology. To define these pathological changes, lung sections were stained with hematoxylin and eosin (H&E) and scored for measures of airway and lung inflammation. IAV infection caused extensive peribronchiolar inflammation, which was present in the majority of airways, with increased cellular infiltrates in the alveolar space and an increased number of inflammatory cells compared with the naive uninfected mice (Fig. 4). However, IAV-infected mice that were treated with MitoTEMPO had reductions in alveolitis and inflammatory cellular infiltrates at both day 3 (Fig. 4) and 5 (Supplementary Figs. S4 and S5) postinfection. Notably, the degree of peribronchiolar inflammation was unchanged with MitoTEMPO treatment.

### MitoTEMPO reduced the number of necrotic and apoptotic cells in lung and BAL

To establish whether MitoTEMPO influenced IAV-induced apoptosis and necrosis of airway and lung macrophages and neutrophils, we examined annexin V and propidium iodide staining in cells isolated from the BAL and lung using flow cytometry. At day 3 p.i, MitoTEMPO treatment significantly attenuated the number of late apoptotic/necrotic macrophages (F480^+^/annexin^−^/PI^+^, Fig. 5A) and neutrophils (Ly6G^+^/annexin^−^/PI^+^; Fig. 5B) in the lung, when compared with the untreated IAV-infected control mice. There was also a significant reduction in the number of late apoptotic/necrotic neutrophils, but not in macrophages from the BAL, in mice treated with MitoTEMPO, when compared with the untreated IAV-infected control mice (Fig. 5C, D).

### MitoTEMPO had no effect on lung humoral and cellular immunity following influenza virus infection

**FIG. 6.**
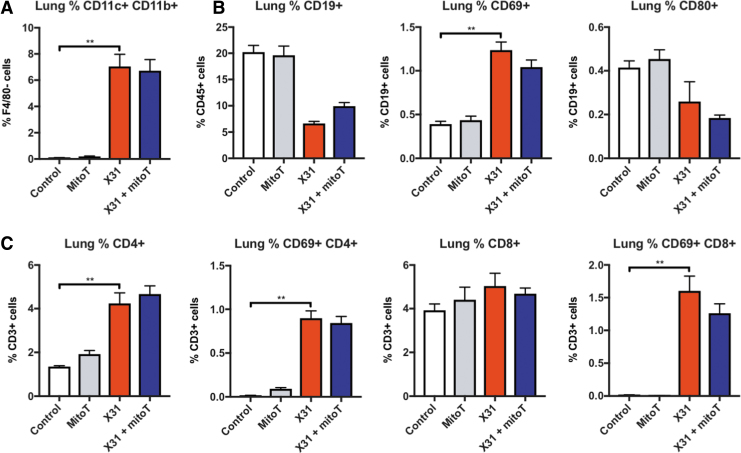
**MitoTEMPO does not alter the adaptive immune response.** C57Bl/6J mice were treated once daily *via* intranasal administration of MitoTEMPO (100 μg) over a 6-day period 1 day before virus infections. Mice were intranasally infected with X31 (10^3^ PFUs) or PBS control. Single cells were isolated from whole lung digests, and multidimensional flow cytometry was used to quantify the relative abundance of **(A)** DCs, **(B)** B cells, and **(C)** T cells. DCs are measured as CD11b^+^ CD11c^+^ cells gated from the F4/80 negative population. T and B cell populations are measured as a percentage of CD3^+^ and CD19^+^ cells, respectively, gated from the CD45^+^ population. Data are expressed as mean ± SEM (Control, *n* = 8; MitoTEMPO, *n* = 8; X31 *n* = 12 X31+mito *n* = 12). Statistical analysis was conducted using one-way ANOVA test followed by Tukey's *post hoc* test for multiple comparisons. Statistical significance was taken where *p* < 0.05 (**p < 0.01). DCs, dendritic cells.

Dendritic cells have important antigen presentation functions that promote T cell immunity. Influenza virus infection caused a marked elevation in myeloid dendritic cells (F4/80^−^CD11C^+^CD11b^+^) in the lung that was unaltered by treatment with MitoTEMPO (Fig. 6A). Also, MitoTEMPO had no effect on myeloid dendritic cells in the spleen (Supplementary Fig. S6). The total B cell populations were reduced with IAV treatment, however, the activated CD69^+^ B cell pool was expanded; MitoTEMPO treatment did not alter this B cell profile at day 5 postinfection (Fig. 6B). There was a decrease in the CD4^+^ T cell population, but activated CD4^+^ T cells were increased at day 5 in IAV-infected mice, which was unchanged with MitoTEMPO treatment, indicating preservation of the adaptive immune response (Fig. 6C). The number of activated CD8^+^ T cells (*i.e.*, CD69^+^) was increased in both IAV-infected mice and MitoTEMPO-treated IAV-infected mice (Fig. 6C).

## Discussion

IAVs cause severe respiratory tract infections that are characterized by the rapid accumulation of inflammatory cells into the lung and oxidative and cellular damage (6, 15, 49, 50). To combat the emergence of drug-resistant viral strains, a possible approach could be to target the host cell mechanisms that promote oxidative stress, viral replication, and inflammation. Indeed, ROS generation by NOX2 oxidase has been implicated in IAV pathology, highlighting that NOX2 oxidase is a potential therapeutic target (22, 37, 44, 49). In this study, we show that mtROS also play an important role in the promotion of the innate immune response to IAV infection in mice. Mice that were intranasally treated with MitoTEMPO to scavenge mitochondrial superoxide displayed a reduction in morbidity to IAV infection and an increase in survival, and this was associated with a marked reduction in airway and lung inflammation. Neutrophil infiltration into the airways was attenuated by MitoTEMPO treatment and the degree of neutrophil apoptosis and necrosis was significantly reduced. MitoTEMPO treatment reduced the amount of secreted IL-1β, but increased the amount of antiviral type I IFN and this was associated with a marked reduction in viral titers and preservation of adaptive immunity. This study identifies an important role for mtROS in the pathogenesis of IAV infection, making this a potential target for therapeutic intervention, to limit viral pathology in humans.

The inhibition of mtROS with MitoTEMPO reduced airway and lung inflammation at both early and later phases of IAV infection, confirming that this is a viable therapeutic strategy. This reduction in airway inflammation in response to IAV infection is similar to that reported for endosomal ROS blockade in NOX2-deficient mice and specific pharmacological NOX2 oxidase inhibitor delivery to endosomes (44). Thus, ROS produced in two distinct subcellular compartments, that is, endosomes and mitochondria, may ultimately have similar effects on the inflammatory response caused by IAV infection, although we are yet to determine if these effects are synergistic or additive. Importantly, our data show that MitoTEMPO treatment has no effect on NOX2 oxidase activity in isolated macrophages, and therefore, the MitoTEMPO is unlikely to be nonspecifically targeting NOX2 oxidase during the initial treatment period before IAV infection. Interestingly, inhibition of ROS in these distinct compartments resulted in both an enhancement in type I IFN levels and a reduction in viral titers. NOX2-derived ROS production in the endosome suppressed TLR7 functionality, most likely by oxidation of cysteine 98 (44). Given that TLR7 is an important pattern recognition receptor that stimulates type I IFN production, the reduction in viral titers due to NOX2 inhibition was attributed to the enhancement in type I IFN. An early increase in type I IFN signaling and suppression of viral load might also account for the reduction in airway and lung inflammation by MitoTEMPO treatment; however, the precise molecular mechanism is yet to be characterized and could also relate to other biologies, including viral infection driving cellular metabolism and ROS production to facilitate energy production and in turn to promote viral replication. Given that mtROS are highly compartmentalized and usually have biological actions within close vicinity of their production, we postulate that a negative regulatory effect of mtROS *per se* on cysteine 98 on TLR7 within the endosomal compartments is unlikely, due to the diffusion limited nature of ROS. However, a possible molecular mechanism for elevated type I IFN is the one proposed by Agod *et al.*, through which mtROS suppress the phosphorylation of IRF7 in the cytosol, which is an important transcription factor for type I IFN expression by TLR7 and TLR9 signaling (1). Therefore, we envisage a complex regulation of type I IFN expression by subcellular-specific ROS, that is, endosome NOX2-derived ROS on C98 of TLR7 directly and mtROS targeting a critical downstream regulator of TLR7 function in IRF7. It is conceivable that targeting both endosomal and mtROS production may be a very effective way of limiting the exacerbated inflammation in response to IAV infection and warrants further investigation. An important consideration is what cell populations are affected by MitoTEMPO treatment to influence type I IFN response. Given that MitoTEMPO treatment did not affect the numbers of macrophages, but reduced neutrophils and eosinophils, future studies should encompass a comprehensive analysis of which lung immune cells, that is, bronchial epithelium, alveolar macrophages, endothelial cells, neutrophils, and eosinophils, are generators of type IFN, which are concomitantly suppressed by mtROS.

Pulmonary inflammation and epithelial damage are common pathological characteristics during IAV infections. In this study, we confirmed that an intranasal challenge with Hkx-31 IAV caused a substantial increase in BALF inflammation, as well as lung alveolitis, bronchiolitis, and extravasation of inflammatory cells into the lungs at day 3 and 5 postinfection. This extensive lung inflammatory response was, however, reduced with MitoTEMPO treatment and was characterized by less alveolitis and generalized inflammatory cell burden, suggesting that mtROS are involved in promoting lung inflammation. The reduction in airway and lung inflammation in response to IAV infection following MitoTEMPO treatment might account for the improvements in body weight. An over active immune response, characterized by exacerbated infiltration of inflammatory cells in response to chemotactic signaling, is a hallmark feature of influenza virus infections, which can exert pathogenic effects such as oxidative damage to cells and surrounding tissue. Neutrophils are one of the first-responder cells during viral respiratory infection and while they are necessary for host recovery (13), can also cause significant tissue damage when in excess. Complete neutrophil depletion in mice resulted in an exacerbated level of pulmonary inflammation and respiratory dysfunction during IAV infection (39, 40), indicating that neutrophils are critical for minimizing viral dissemination and disease severity (14). However, partial suppression of neutrophil infiltration into the lung limits viral pathology, due to a reduction in neutrophil-dependent excessive ROS and protease production. In this study, the blockade of mtROS resulted in an ∼50% suppression of neutrophil migration into the virus-infected airways, as well as a reduction in neutrophil apoptosis and necrosis. It also resulted in a significant reduction in BALF ROS production that is attributed to NOX2 oxidase. Given that the neutrophil is the cell type within the population of cells in the BALF that generate by far the greatest amount of ROS, the ∼50% reduction in neutrophil numbers most likely accounts for the ∼50% reduction in BALF ROS production. MitoTEMPO treatment also resulted in an attenuation of eosinophil numbers in the airways, which has been associated with a reduction in viral burden in asthma models (31) and thus might be protective during IAV infection. Our study also showed that MitoTEMPO treatment did not modify macrophage or NK cell recruitment at day 3, and thus, it is likely that critical immune functions of these cells are preserved during the early and peak phases of innate immunity. Moreover, the effects of MitoTEMPO appear to be localized to the lung as it did not modify systemic inflammation measured in the blood. Collectively, these observations indicate that mitochondria-targeted ROS inhibition effectively reduces the number of infiltrating immune cells into the lung, particularly neutrophils, which may provide protection by attenuating the detrimental effects of an exacerbated inflammatory response.

NLRP3 inflammasome activation drives IL-1β production and other proinflammatory cytokines by phagocytic and nonphagocytic cells at the site of injury during IAV infection. This acute cytokine response is fundamental in regulating leukocyte migration, cell survival, and apoptosis. However, inflammasome activation during IAV infection can potentially give rise to two opposing outcomes. An early activation of the inflammasome appears to be critical for protecting the host from IAV-induced morbidity and mortality (38). However, persistent and late-stage inflammasome activity exacerbates the inflammatory response and increases the pathogenesis. Inflammasome activation requires ROS and the mitochondria have been touted as one of the major sources of these ROS (38). Our study confirmed that mtROS are critical for driving the inflammasome at the early stages of infection. Indeed, MitoTEMPO treatment significantly decreased the protein levels of IL-1β in the airways. Interestingly, and in striking contrast, MitoTEMPO *increased* IL-1β mRNA expression in the airways. The inverse correlation between IL-1β gene expression and protein levels may be attributed to an ROS-dependent suppression of NF-κB signaling and thereby reducing IL-1β transcription (51). Also, consistent with our observation are studies showing that pharmacological inhibitors of ROS reduce the secretion of mature IL-1β but not the biosynthesis of pro-IL-1β (7). From the observations that MitoTEMPO decreased IL-1β protein, it is anticipated that scavenging of mtROS with MitoTEMPO should exacerbate the IAV-induced pathology by decreasing inflammasome activation. This phenotype in the presence of MitoTEMPO should mimic that due to NLRP3 inhibition, and indeed, our findings with MitoTEMPO are similar to NLRP3 inhibition—that is, there is a reduction in IL-1β and a decrease in neutrophil recruitment and airway inflammation. However, we have noted some critical differences. That is, MitoTEMPO treatment resulted in an ameliorated body weight loss and thus a reduction in morbidity and mortality. By contrast, early NLRP3 inhibition resulted in an exacerbated weight loss and increase in mortality. These contrasting outcomes of early mtROS inhibition *versus* early NLRP3 inhibition might be attributed to the concomitantly enhanced type I IFN production and reduced viral titers that we observed with MitoTEMPO treatment. NLRP3 inhibition did not result in alterations in viral titers (41, 42), and thus, MitoTEMPO treatment has the capacity to not only reduce IL-1β-dependent neutrophilia but also reduce the viral burden.

It has been reported that hypercytokinemia contributes to severe IAV infections and that this predicts IAV fatalities (10, 48). In the present study, IAV infection resulted in a significant inflammatory response at day 3 that persisted to day 5, as characterized by elevations in airway neutrophils and inflammatory cells. With prolonged mtROS inhibition, we demonstrate significant attenuation in airway and lung inflammation, viral titers, as well as the expression of several proinflammatory (TNF-α, IL-6, IL-1β), antiviral (IFN-β) and chemotactic (IL-17A, CXCL2, CCL2) cytokines at day 5 postinfection. These findings demonstrate that an early elevation in type I IFN and a reduced viral burden due to mtROS inhibition promote a more rapid overall resolution of inflammation in the airways and lungs and that this is associated with an improvement in the health status of the mice. An important question is what types of cells are involved in the production of these cytokines at this day 5 time point, which are producing significantly less proinflammatory cytokines. Several immune cells are involved in generating cytokines at the later stages of IAV infection. Inflammatory cells, including alveolar macrophages and monocytes, mainly produce TNF-α, IL-6, IFN-β, and chemokines (CCL3 and CXCL2); neutrophils produce TNF-α, IL-6, IL-1β, and IFN-γ; and T cells are sources of IFN-γ and IL-17A (3, 45), although neutrophils are also potential sources of IL-17A. Given that CD4^+^ and CD8^+^ T cell numbers were unaffected by MitoTEMPO, it is tempting to speculate that their production of IFN-γ and IL-17A is not affected by MitoTEMPO, but that the reduced number of neutrophils is accounting for the reduction in IL-17A. Given that we observed significant reductions in macrophages and neutrophils, we would suggest that this is likely to explain the reductions in TNF-α, IL-6, IFN-β, and CCL3 and CXCL2.

In addition, suppression of mtROS is highly likely to modify the redox and metabolic profile of macrophages, and thus, the levels of pro- and antiviral cytokines released in response to IAV infection. This certainly warrants a comprehensive analysis in future studies.

Antigen presenting cells form an integral part of bridging the innate and adaptive immune response during influenza virus infection (9, 47). Emerging evidence implicates that mitochondria serve as signaling platforms to modulate adaptive immunity, in particular in the proliferation, activation, and apoptosis of T cells (8, 35, 52). Dendritic cells have specialized antigen presentation functions that are involved in activating T cell immunity, specifically CD4^+^ and CD8^+^ responses (4, 36). Several recent studies have demonstrated mtROS, in particular H_2_O_2_ regulates the induction of CD8^+^ T cells and the antigen presentation by plasmacytoid DCs to CD8^+^ T cells (29). These observations do not appear consistent with our findings, as we observed no alterations in the IAV-induced activation of both lung and splenic CD8^+^ and CD4^+^ T cell responses with MitoTEMPO treatment at the early and later stages of infection. However, there are some important points that need to be raised in regard to mtROS and T cell activation, and plasmacytoid dendritic cell (pDC)-dependent T cell activation. It appears that there is a high degree of redundancy in the mtROS-dependent activation of Ag cross-presentation (29). This is evident in studies that have utilized mtROS scavengers at different concentrations resulting in graded reductions in mtROS (29). For example, partial suppression of mtROS with S3QEL2, a mitochondrial-targeted antioxidant, had no effect on the cross-presentation capacity of pDC (29). However, only when there was an almost complete reduction in mtROS by S3QEL2 was there evidence for a reduction in pDC cross-presentation. The findings of our study are thus consistent with such previous work. Indeed, we showed that partial suppression of mtROS in macrophages and other CD45^+^ cells in the airways and lungs had no effect on the activation of both CD8^+^ and CD4^+^ T cells. Furthermore, we also have evidence that naive and activated B cells were unaltered with MitoTEMPO treatment in the lung following IAV infection, suggesting overall that partial suppression of mtROS does not impact the critical components of adaptive and humoral immune responses. It is highly likely that mtROS have a multitude of effects on both innate and adaptive immune responses following not only viral infections such as influenza but also to other pathogens such as bacteria and fungi. As for the road-map for future initiatives in this mtROS field, which is at present in its infancy, it will be critical to establish the following. (i) What are the culprit ROS (*i.e.*, superoxide *vs.* H_2_O_2_ *vs.* peroxynitrite) and where are their molecular targets? (ii) The threshold of ROS production for regulation of inflammasome activation *versus* Ag presentation and T cell activation. (iii) The potential crosstalk between mtROS and other sources of ROS such as endosomal NOX2 in the pathogenesis of influenza infection.

In conclusion, a partial suppression of mtROS generation with MitoTEMPO facilitates a more effective innate immune response to IAV, which limits lung pathology, improves survival, while preserving adaptive immunity and facilitating viral clearance. These findings provide evidence for the pharmacological targeting of mtROS with intranasal delivery of mitochondrial antioxidants as a means to reduce the morbidity and mortality of IAV infection. Intranasal delivery of MitoTEMPO to effectively reduce influenza immunopathology presents a favorable means of drug delivery, which enhances the translation potential for this technology. Of importance here is that suppressing disease severity is unlikely to be due to alterations in systemic effects, since the inhibition stems from direct activity within the lungs—that is, preabsorptively. Indeed, intranasal delivery most likely minimizes any off-target toxicology that might arise from systemic administration and, in doing so, allows for higher dosing with reduced toxicity.

## Experimental Procedures

### Viruses

The IAV Hkx31 (H3N2) strain was provided by Prof Patrick Reading (Department of Immunology and Microbiology, University of Melbourne, the Peter Doherty Institute for Infection and Immunity). The virus was provided in phosphate-buffered saline (PBS) and stored at −80°C until used. On the day of infection, the virus was thawed and diluted to an appropriate concentration.

### Animal ethics statement

All mouse experimentations described in this article were approved by the Animal Experimentation Ethics Committee of RMIT University and conducted in compliance with the guidelines of the National Health and Medical Research Council (NHMRC) of Australia on animal experimentation.

### *In vivo* infection with IAV and intranasal delivery of pharmacological agents

Eight- to 12-week-old male C57Bl/6J mice were anesthetized by isoflurane inhalation and infected intranasally with one of two doses of IAV. The first dose of IAV was 10^4^ PFUs of Hk-x31 in a 50 μL volume, diluted in PBS. This was to perform analyses at day 3, which is the peak of the early innate immune response. The second dose of IAV was 10^3^ PFUs of Hk-x31 in a 50 μL volume, diluted in PBS to perform the day 5 analyses. In preliminary experiments, we noticed that mice under repeated doses of anesthetic lost slightly more weight than usual, following IAV infection. Due to the constraints of our animal ethics, mice are to be euthanized if they lose more than 20% of their body weight. Since we wanted to test both early and later stages of infection (day 3 and 5 postinfection), the day 5 postinfection required a lower dose of virus (10^3^ PFUs) to prevent a greater than 20% weight loss and to allow for a full characterization of innate and adaptive immune system responses. Mice were euthanized at day 3 or 5 following IAV infections.

In specific experiments, anesthetized mice were treated once daily *via* intranasal delivery with either PBS (control) or MitoTEMPO (100 μg) 1 day before infection with Hk-x31 and every day thereafter for 4 days. The treatment strategy is more clearly depicted in the schematic provided (Supplementary Fig. S7). This particular dose and route of administration has not previously been explored and was decided by using a midrange dose based on studies that have used it *via* osmotic minipumps and intraperitoneal injections, in models of hypertension (12). Evidently, these studies achieved a partial inhibition of mtROS in intact cells following MitoTEMPO treatment. In addition, mitochondrial respiration was measured in isolated mitochondria and there was an improvement of respiratory coupling as a result of diminished levels of mtROS following MitoTEMPO treatment, indicative of reversing mitochondrial dysfunction by reducing electron leakage (24). In our model, MitoTEMPO was administered 1 day before infection and then once daily for four consecutive days, with endpoint analyses taken at day 3 and 5 postinfection. The dose of MitoTEMPO was able to effectively reduce mtROS, but did not have any adverse effects when used in the absence of IAV on any of the endpoint measurements, including airway and lung inflammation, body weight, or ROS production, which made it an appropriate dose to determine the ability of the drug to alleviate morbidity without causing any toxicity to the animals.

### Airway inflammation and differential cell counting

Mice were killed by intraperitoneal infection with a mixture of ketamine/xylazine (360 mg/kg), and BAL differential cell counting performed as we have previously described (49).

### Primary cell isolation

Alveolar macrophages were isolated by lung lavage, from 8- to 12-week-old male C57Bl/6J mice. A thin shallow midline incision from the lower jaw to the top of the rib cage was made and the larynx was separated to expose the top of the trachea. The layer of smooth muscle covering the trachea was removed, a small incision made, and a sheathed 21-gauge needle was inserted into the lumen. The lungs were lavaged three times with 300–400 μL of PBS (Sigma). Cells were kept at 37°C under humidified conditions with 5% CO_2_ and 95% air.

### L-O12-enhanced chemiluminescence for the detection of NOX2 oxidase-dependent ROS

ROS production was quantified using L-O12-enhanced chemiluminescence. Cells isolated from the BAL were seeded into a 96-well OptiView plate (5 × 10^4^ cells/well) with Dulbecco's modified Eagle's medium (Thermo Fisher) containing 4.5 g/L of glucose, 110 mg of sodium pyruvate, and 10% fetal bovine serum (FBS; Sigma-Aldrich), and allowed to adhere for 3 h before starting the assay. Cells were washed in Krebs-HEPES buffer at 37°C and then exposed to a Krebs-HEPES buffer containing L-O12 (10^−4^ *M*) in the absence (*i.e.*, basal ROS production) or presence (stimulated ROS production) of the protein kinase C and NADPH oxidase activator phorbol dibutyrate (10^−^6 *M*). The same treatments were performed in blank wells (*i.e.*, with no cells), which served as controls for background luminescence. All treatment groups were performed in triplicate. Photon emission (relative light units/s) was detected using the BMG Labtech microplate reader (CLARIOstar, Germany) and recorded from each well for 1 s over 60 cycles. Individual data points for each group were derived from the average values of the three replicates minus the respective blank controls.

### Quantification of mRNA by qPCR

Ten milligrams of crushed lung tissue was used to extract total RNA using an RNeasy mini kit (Qiagen). Synthesis of cDNA was performed using the High-Capacity cDNA RT kit (P/N4322171; Life Technologies, Foster City, CA) using 1.0–3.0 μg total RNA. qPCR was carried out using the TaqMan Fast advanced Master Mix (Life Technologies) or SYBR Green PCR Master Mix (Life Technologies) and analyzed on the QuantStudio 7 Flex Real-Time PCR system (Life Technologies). The PCR primers for TNF-α, IL-1β, IFN-β, IL-6, CCL3, CXCL2, IL-17A, and IFN-γ were included in the Assay-On-Demand Gene Expression Assay Mix (Life Technologies). In addition, custom-designed forward and reverse primers of the segment 3 polymerase (PA) of IAV were used to measure viral titers. The PCR program run settings were as follows: 50°C for 2 min, followed by 95°C for 1 h, and then 95°C for 15 s + 60°C for 60 s + plate read (40 cycles). For FAST ADVANCED MASTER MIX, the program settings were as follows: 50°C for 2 min, 95°C for 2 min, 95°C for 1 s, and 60°C for 20 s + plate read (40 cycles). Quantitative values were obtained from the threshold cycle (Ct) number. Target gene expression was normalized against glyceraldehyde 3-phosphate dehydrogenase mRNA expression for each sample, and data were expressed relative to the naive control group.

### Enzyme-linked immunosorbent assay and multiplex immunoassay

The amount of IL-1β protein was measured in BALF using enzyme-linked immunosorbent assays and performed using commercially available kits (R&D System, Minneapolis, MN). First, 50 μL of BALF was added to the precoated 96-well plate and allowed to incubate for 2 h at room temperature, and was washed five times with 400 μL of washing buffer. Next, 100 μL of the detection antibodies was added and allowed to incubate for 2 h, followed by the washing step as previously stated. Fifty microliters of streptavidin-horseradish peroxidase (catalog No. DY998, diluted according to the directions on the vial label; R&D Systems) was added to each well. The plate was covered with an adhesive seal and incubated for 30 min at room temperature in the dark, then washed and aspirated as above, before the addition of the substrate solution and finally the stop solution. The 96-well plate was read on a microtiter plate reader (Molecular Devices, Sunnyvale, CA) at a wavelength of 450 nm. Cytokine titers in the samples were determined by plotting the optical densities, using a four-parameter fit for the standard curve and expressed in pg/mL.

### Histology

The left lung was dissected from mice and fixed in neutral buffered formalin (10%) for 24–48 h. Following this, the lung tissue was processed in paraffin wax, and longitudinal 3–4 μm sections cut and stained with H&E. Slides were scanned by light microscopy and uploaded to the Aperio microscope scanner (Leica Biosystems, Nussloch, Germany). Histology was performed by the Department of Histology (Monash University, Clayton, Australia) and analyzed blind by two separate investigators using an Aperio ImageScope.

### Flow cytometry

Whole lung was finely minced with scissors and enzymatically digested using Liberase (Sigma). Single-cell suspensions were prepared from homogenized lung, BAL, and spleen by straining through a 40 μ*M* mesh filter. The red blood cells were lysed with lysis buffer (ACK lysis buffer) and the white blood cells stained with respective fluorescent-labeled anti-mouse antibodies for flow cytometric analysis: CD45 (30-F11), TCRγδ (GL3; BioLegend), CD3 (145–2C11), CD8 (53–6.7), CD44 (IM7), CD69 (H1.2F3), CD19 (1D3), NK1.1 (PK136) (BD Bioscience), CD80 (16-10A1), CD4 (GK1.5), CD11b (M1–70), CD11c (N418), F4/80 (BM8), and Ly6G (1A8; Invitrogen). Within each antibody cocktail mixture, cells were incubated with CD16/32 (2.4G2) to block Fc-mediated adherence of the antibodies. Cell viability was determined by the LIVE/DEAD Fixable Violet Dead Cell Stain Kit (Invitrogen). Total mtROS were assessed using the fluorescent dye-based MitoSOX™ Red with the absorption/emission: 510/580 nm. Markers for apoptosis and necrosis involved staining with Annexin-V (BioLegend) and PI (Invitrogen). A minimum of 100,000 events were acquired for each sample on the FACSAria II (BD Biosciences) and data analysis carried out using FlowJo software. Manual clustering of multidimensional flow cytometry was guided by isotype controls and/or untreated samples.

### Chemicals

MitoTEMPO (Cat No. 1334850-99-5; Sigma) was dissolved in PBS and prepared as a stock solution of 1 mg/mL and stored at −20°C until use. FBS (Sigma) was stored in 50 mL aliquots at −20°C. MitoSOX (Thermo Fisher Scientific) was dissolved in DMSO (100%) at stock solutions of 1 μg/mL and stored in aliquots of 2 μL at −20°C.

### Statistical analysis and image analysis

All statistical tests were performed using GraphPad Prism (GraphPad Software Version 7.0, San Diego, CA). *p* < 0.05 was taken to indicate significance. For survival curves, a Kaplan–Meier analysis was performed.

## Supplementary Material

Supplemental data

Supplemental data

Supplemental data

Supplemental data

Supplemental data

Supplemental data

Supplemental data

## Abbreviations Used

BAL: bronchoalveolar lavage
BALF: bronchoalveolar lavage fluid
FBS: fetal bovine serum
H&E: hematoxylin and eosin
IAV: influenza A virus
mtROS: mitochondrial reactive oxygen species
NADPH: nicotinamide adenine dinucleotide phosphate
NLRP3: NACHT, LRR, and PYD domain-containing protein 3
PA: polymerase
PBS: phosphate-buffered saline
PDB: phorbol dibutyrate
pDC: plasmacytoid dendritic cell
PFU: plaque forming unit
PI: propidium iodide
qPCR: quantitative polymerase chain reaction
ROS: reactive oxygen species
SEM: standard error of the mean
