# Supplementary material for: Mitochondrial Reactive Oxygen Species Contribute to Pathological Inflammation During Influenza A Virus Infection in Mice
Source: Antioxid Redox Signal. 2020 Mar 24;32(13):929–42. doi: 10.1089/ars.2019.7727 (PMC7104903; doi:10.1089/ars.2019.7727)
Supplement: Supplemental data [file Supp_Fig1.pdf]

Supplementary Data

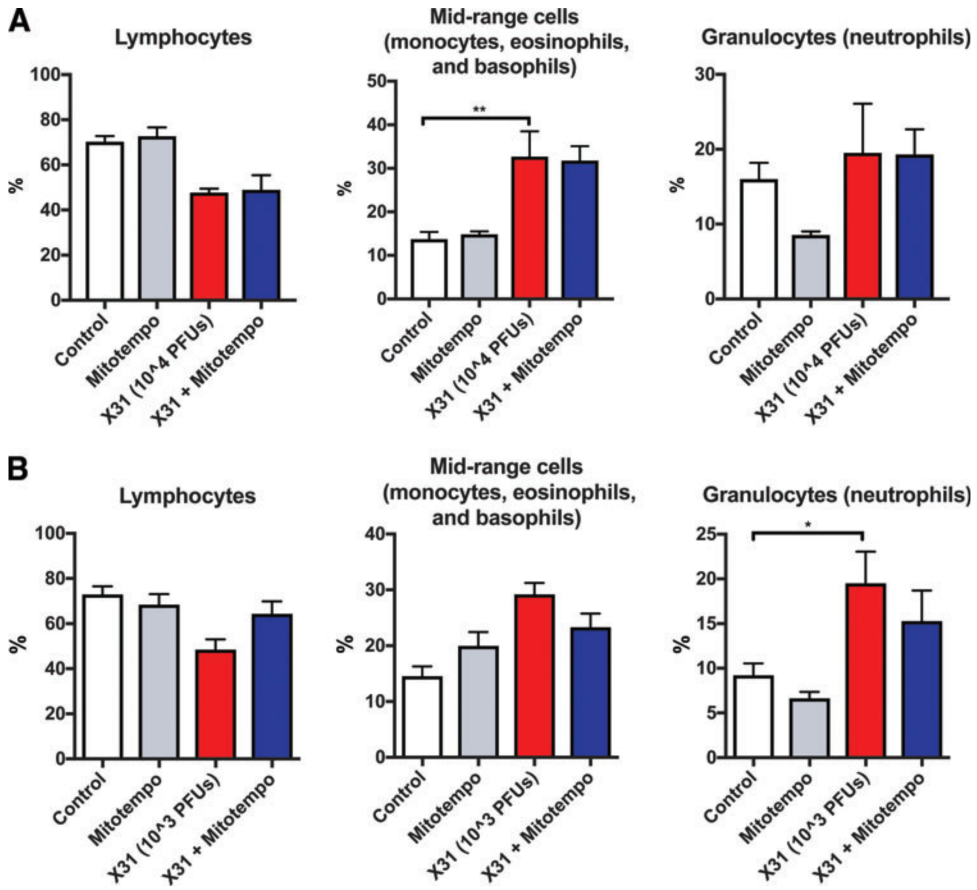

SUPPLEMENTARY FIG. S1. MitoTEMPO has no effect on systemic inflammation at day 3 (A) or day 5 (B) postinfluenza infection. Mice were treated once daily via intranasal administration of MitoTEMPO (100 μg) over a 4–6-day period 1 day before virus infections. Mice were intranasally infected with X31 (10^4 PFUs in **A** or 10^3 PFUs in **B**) or PBS control. Measurements of lymphocytes, granulocytes, and midrange cells (monocytes, eosinophils, and basophils) were recorded in whole blood using the CELL-DYN Emerald 22 Hematology Analyzer (Control, $n=8$; MitoTEMPO, $n=8$; X31 $n=16$ X31+mito $n=16$). Statistical analysis was conducted using one-way ANOVA test followed by Tukey's *post hoc* test for multiple comparisons. Statistical significance was taken where $*p<0.05$ and $**p<0.01$. PBS, phosphate-buffered saline; PFU, plaque forming unit.
