# Supplementary material for: Mitochondrial Reactive Oxygen Species Contribute to Pathological Inflammation During Influenza A Virus Infection in Mice
Source: Antioxid Redox Signal. 2020 Mar 24;32(13):929–42. doi: 10.1089/ars.2019.7727 (PMC7104903; doi:10.1089/ars.2019.7727)
Supplement: Supplemental data [file Supp_Fig2.pdf]

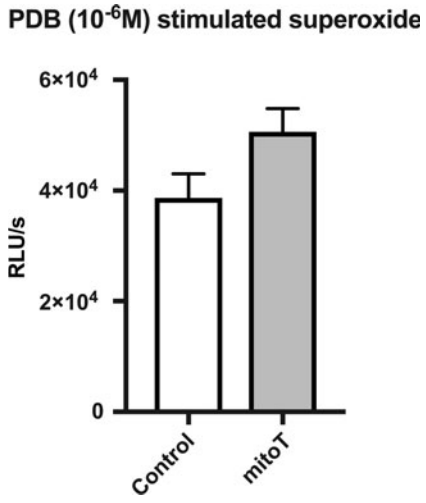

SUPPLEMENTARY FIG. S2. MitoTEMPO has no effect on NOX2 oxidase activity in isolated macrophages. The NOX2 oxidative burst was measured by L-012-enhanced chemiluminescence in the macrophage cell line RAW 264.7 cells pretreated with MitoTEMPO (5 m*M*) for 1 h. RLU/s are represented as mean ± SEM of $n = 9$ experiments. RLU, relative light units; SEM, standard error of the mean.
