# Supplementary material for: Mitochondrial Reactive Oxygen Species Contribute to Pathological Inflammation During Influenza A Virus Infection in Mice
Source: Antioxid Redox Signal. 2020 Mar 24;32(13):929–42. doi: 10.1089/ars.2019.7727 (PMC7104903; doi:10.1089/ars.2019.7727)
Supplement: Supplemental data [file Supp_Fig3.pdf]

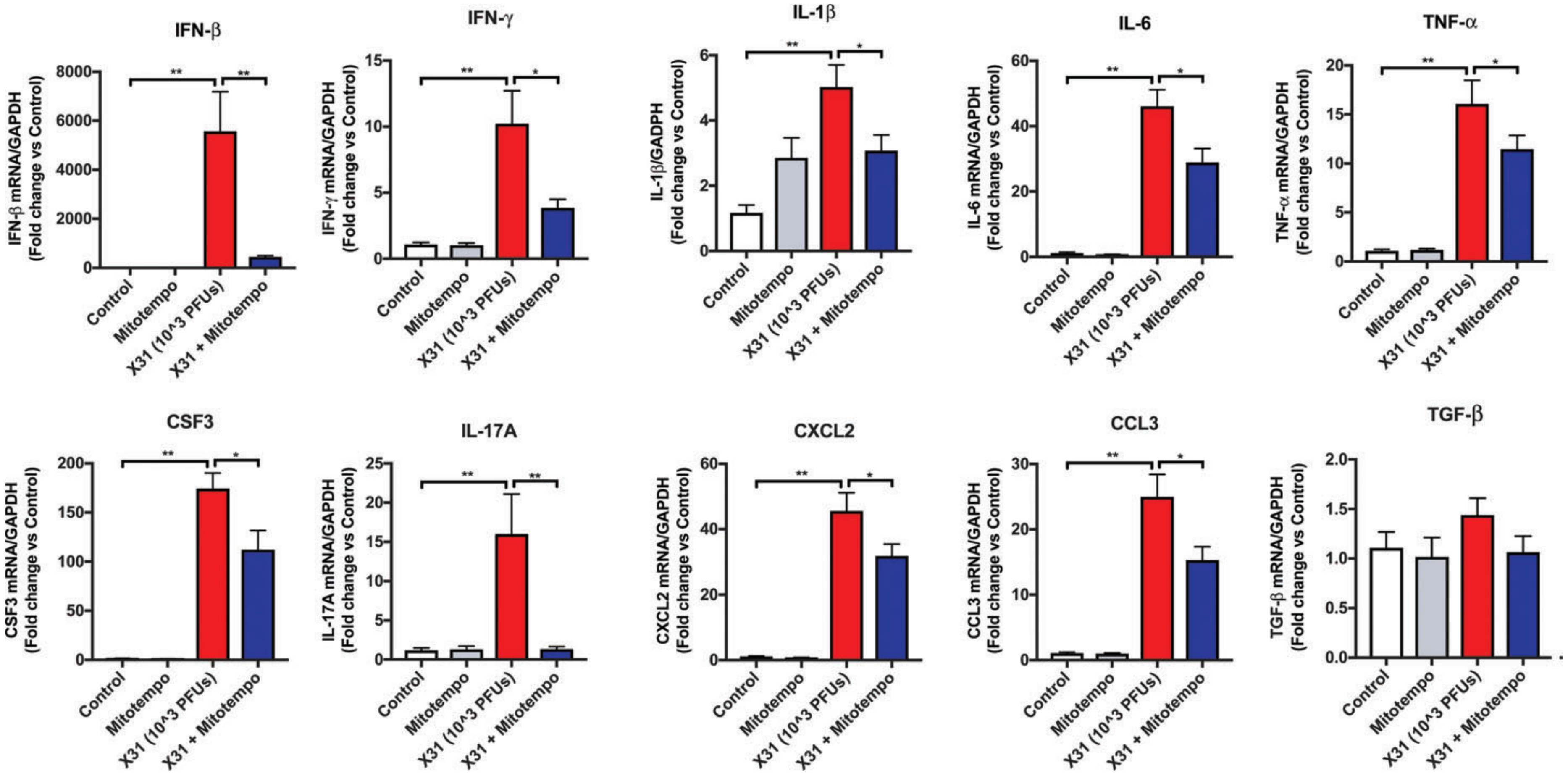

SUPPLEMENTARY FIG. S3. Marked suppression of proinflammatory and antiviral cytokine expression with MitoTEMPO treatment at day 5 postinfection. Mice were infected with Hkx-31 at (10^3 PFUs) or PBS (control) *via* intranasal administration. Animals were treated once daily via intranasal administration of MitoTEMPO (100 μg) over a 6-day period 1 day before virus infection for measurements at day 5 p.i. qPCR was used to quantify expression of cytokines and chemokines: IFN-β, IFN-γ, IL-1β, IL-6, TNF-α, IL-17A, CXCL2, and CCL3. Responses are relative to GAPDH and then expressed as a fold-change above naive controls. Data are expressed as mean ± SEM (Control, $n = 6$–8; MitoTEMPO, $n = 6$–8; X31 $n = 10$ X31+mito $n = 12$–14). Statistical analysis was conducted using one-way ANOVA test followed by Tukey's *post hoc* test for multiple comparisons. Statistical significance was taken where $p < 0.05$ (*$p < 0.05$ and **$p < 0.01$). qPCR, quantitative polymerase chain reaction.
