# Supplementary material for: Mitochondrial Reactive Oxygen Species Contribute to Pathological Inflammation During Influenza A Virus Infection in Mice
Source: Antioxid Redox Signal. 2020 Mar 24;32(13):929–42. doi: 10.1089/ars.2019.7727 (PMC7104903; doi:10.1089/ars.2019.7727)
Supplement: Supplemental data [file Supp_Fig4.pdf]

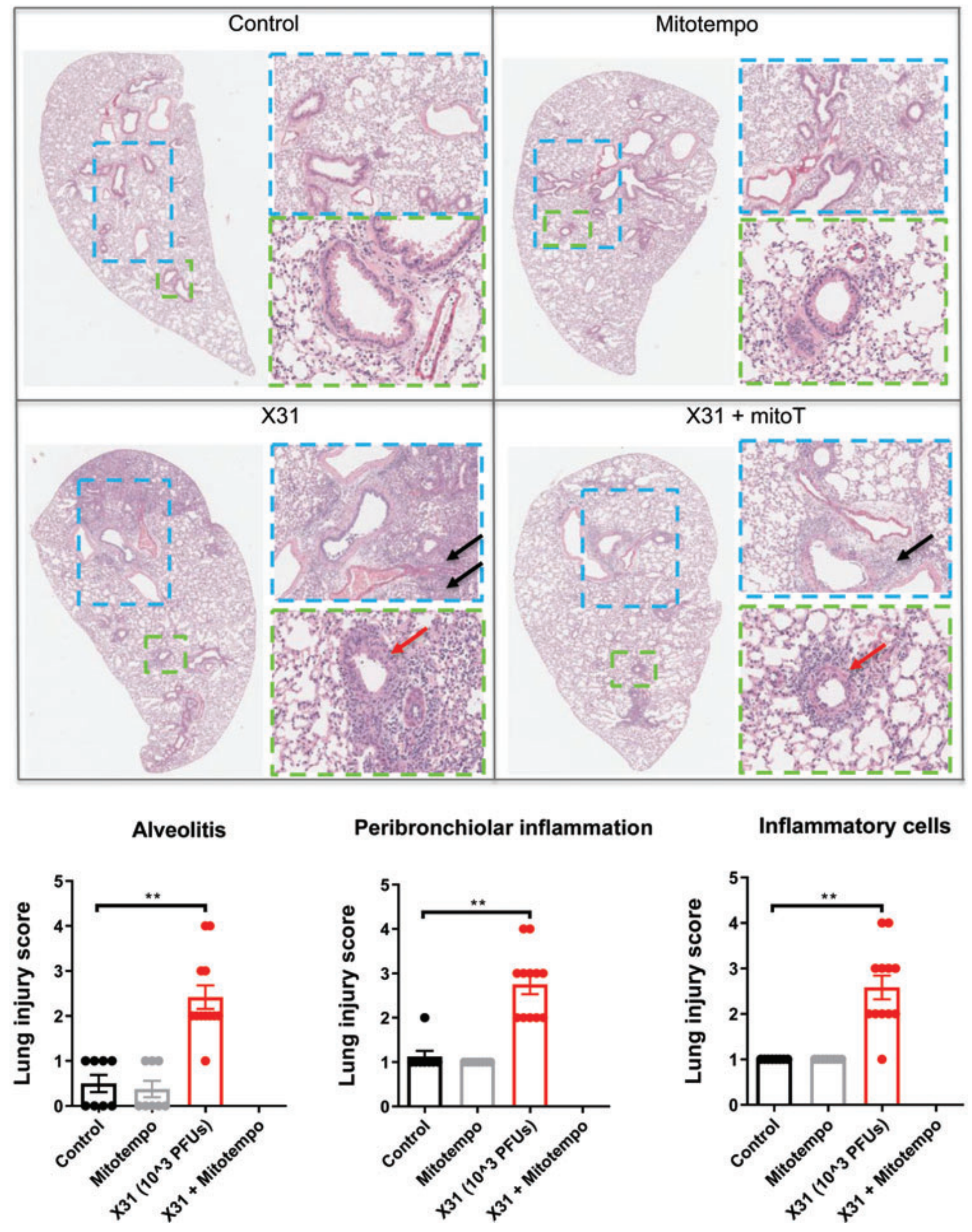

SUPPLEMENTARY FIG. S4. MitoTEMPO relieves lung histopathological changes in Hkx-31-infected mice at day 5. Histopathological analysis of lungs from WT C57Bl/6J mice treated once daily via intranasal administration of Mito-TEMPO (100 μg) over a 6-day period 1 day before virus infections. Mice were intranasally infected with X31 (10^3 PFUs) or PBS (control). Representative images displaying the inflammation in lung were sectioned longitudinally following H&E staining at day 5 postinfection. Each sample was scored blindly from 0 to 5 for each individual mouse (higher numbers indicate increased disease severity) from two independent assessors. Sections were scored for alveolitis (*black arrows*), inflammatory cell infiltrate, and peribronchiolar inflammation (*red arrows*). Representative images are presented at three different magnifications (1× [clear], 3× [*blue*], 6× [*green*]). Data are expressed as mean ± SEM (Control, $n = 6$–8; Mito-TEMPO, $n = 6$–8; X31 $n = 10$ X31+mito $n = 12$–14). Statistical analysis was conducted using one-way ANOVA test followed by Tukey's *post hoc* test for multiple comparisons. Statistical significance was taken where $p < 0.05$ (**$p < 0.01$).
