# Supplementary material for: Mitochondrial Reactive Oxygen Species Contribute to Pathological Inflammation During Influenza A Virus Infection in Mice
Source: Antioxid Redox Signal. 2020 Mar 24;32(13):929–42. doi: 10.1089/ars.2019.7727 (PMC7104903; doi:10.1089/ars.2019.7727)
Supplement: Supplemental data [file Supp_Fig5.pdf]

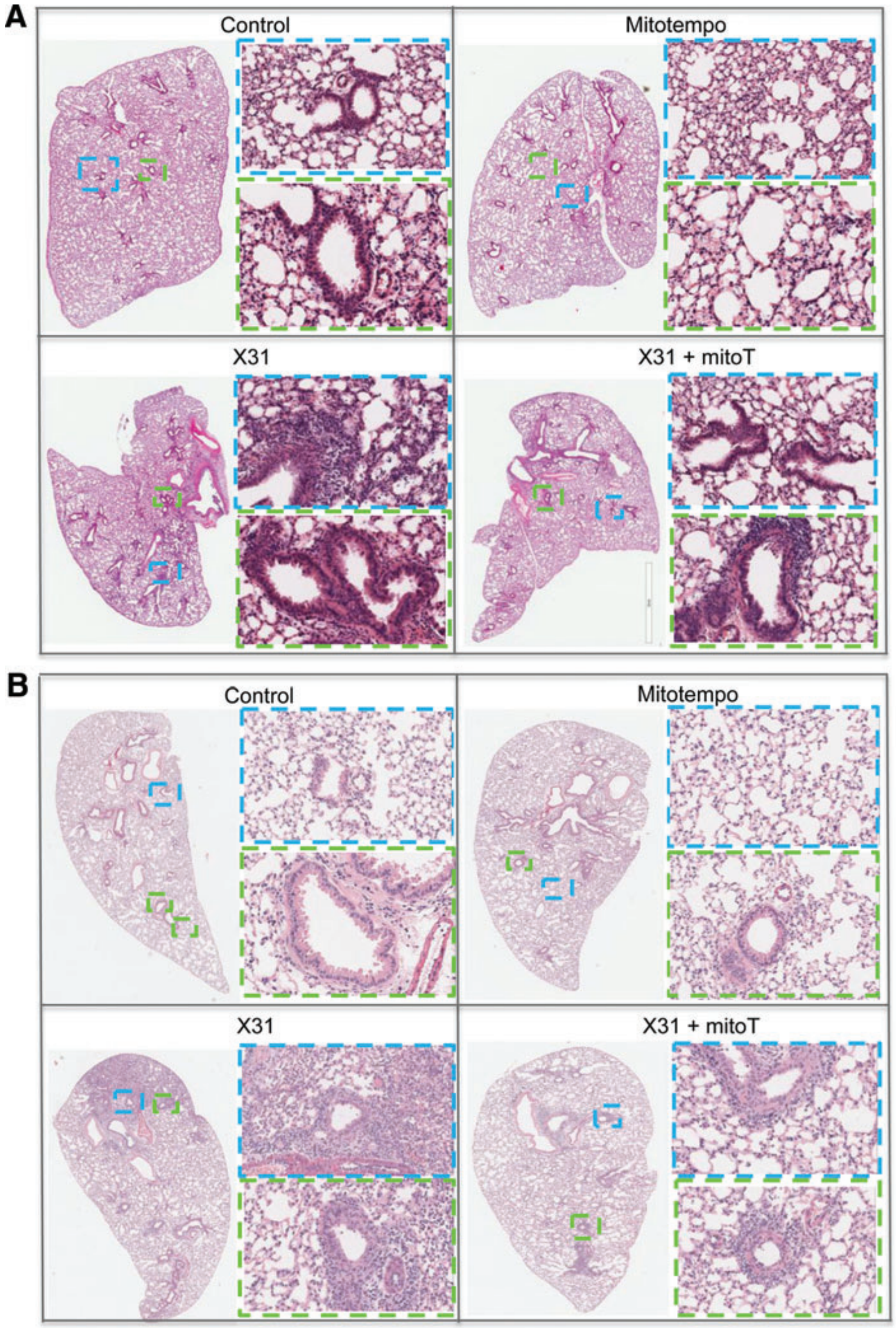


**SUPPLEMENTARY FIG. S5. Lung histopathological changes in Hkx-31-infected mice at day 3 and 5 postinfection.** Histopathological H&E analysis of lungs from WT C57Bl/6J mice treated once daily via intranasal administration of MitoTEMPO (100 $\mu$g) over a 4–6-day period 1 day before virus infections. Mice were intranasally infected with X31 ($10^3$–$10^4$ PFUs) or PBS (control) and culled for assessment at **(A)** day 3 p.i or **(B)** day 5 p.i. Representative images are at three different magnifications (1×[clear], 10×[*blue*], 20×[*green*]). Data are expressed as mean ± SEM (Control, $n$ = 6–8; MitoTEMPO, $n$ = 6–8; X31 $n$ = 10 X31+mito $n$ = 12–14).
