# Supplementary material for: Mitochondrial Reactive Oxygen Species Contribute to Pathological Inflammation During Influenza A Virus Infection in Mice
Source: Antioxid Redox Signal. 2020 Mar 24;32(13):929–42. doi: 10.1089/ars.2019.7727 (PMC7104903; doi:10.1089/ars.2019.7727)
Supplement: Supplemental data [file Supp_Fig6.pdf]

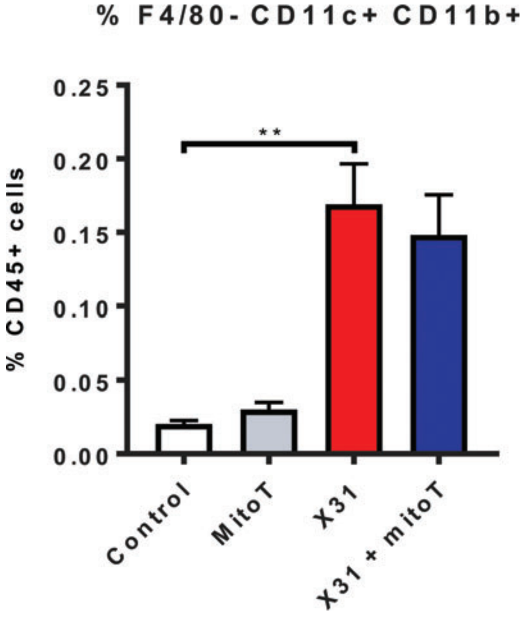

SUPPLEMENTARY FIG. S6. While IAV increased the number of splenic dendritic cells, MitoTEMPO treatment did not alter this dendritic cell population in the spleen. C57Bl/6J mice were treated once daily via intranasal administration of MitoTEMPO (100 μg) over a 6-day period 1 day before virus infections. Mice were intranasally infected with X31 (10^4 PFUs) or PBS control. Single cells were isolated from spleen and multidimensional flow cytometry was used to quantify the relative abundance of myeloid DCs. DCs were measured as $CD11b^+$ $CD11c^+$ cells gated from the F4/80 negative population. Data was expressed as mean ± SEM (Control, $n = 8$; MitoTEMPO, $n = 8$; X31 $n = 12$ X31+mito $n = 12$). Statistical analysis was conducted using one-way ANOVA test followed by Tukey's *post hoc* test for multiple comparisons. Statistical significance was taken where $p < 0.05$ (**$p < 0.01$). DCs, dendritic cells; IAV, influenza A virus.
