# Supplementary material for: Mitochondrial Reactive Oxygen Species Contribute to Pathological Inflammation During Influenza A Virus Infection in Mice
Source: Antioxid Redox Signal. 2020 Mar 24;32(13):929–42. doi: 10.1089/ars.2019.7727 (PMC7104903; doi:10.1089/ars.2019.7727)
Supplement: Supplemental data [file Supp_Fig7.pdf]

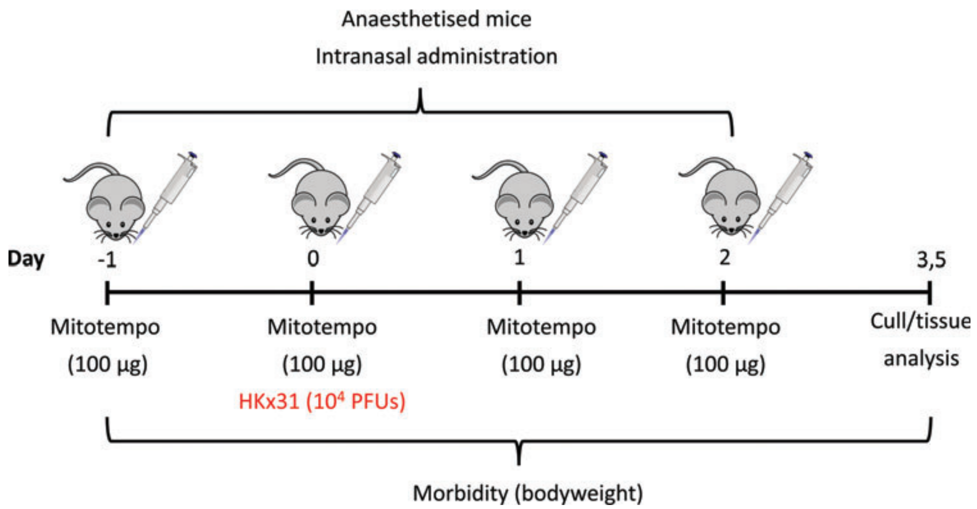

SUPPLEMENTARY FIG. S7. Treatment strategy. Anesthetized mice were treated once daily *via* intranasal delivery with either PBS (control) or MitoTEMPO (100 μg) 1 day before infection with Hk-x31 (10^3 or 10^4 PFUs) and every day thereafter for 4 days and culled for endpoint analyses at day 3 and 5 postinfection.
